# Classical vs. New Mini-Window Medialization Laryngoplasty Method in Glottic Insufficiency

**DOI:** 10.22038/ijorl.2025.78056.3622

**Published:** 2025

**Authors:** Aslan Ahmadi, Nadia Goudarzi, Mohammad Mahdi Salem, Mojtaba Maleki Delarestaghi, Fatemeh Jahanshahi

**Affiliations:** 1 Ear-Nose-Throat and Head and Neck Surgery Research Center, Rasul-e-Akram Hospital, Iran University of Medical Sciences, Sattarkhan street, Tehran, Iran.; 2 ENT and Head and Neck Research Center and Department, The Five Senses Institute, Iran University of Medical Sciences, Tehran, Iran; Skull Base Research Center, The Five Senses Institute, Iran University of Medical Sciences, Tehran, Iran.; 3 Student Research Committee, Faculty of Medicine, Iran University of Medical Sciences, Tehran, Iran.

**Keywords:** Otolaryngology, Medialization laryngoplasty, Glottis; Gore-Tex Implants, Voice Quality, Vocal Cord Paralysis

## Abstract

**Introduction:**

True vocal cord failure (glottis) refers to the inability or failure of the larynx structure and vocal cords to produce sound. One of the optimal techniques for repairing this lesion is medialization laryngoplasty, which restores sound strength, continuity, and quality. Another technique is to create a Mini window in the periphery of false vocal cords. This study compared the effectiveness of medialization laryngoplasty with Gore-tex in two classic medialization laryngoplasty techniques and the Mini window method in the periphery of false vocal cords.

**Materials and Methods:**

A total of 30 participants who had unilateral vocal cord paralysis due to various causes, such as surgery, metastasis, and intubation, were studied. After obtaining informed consent from patients, 15 participants were assigned to each group (classic or mini-window medialization laryngoplasty). Voice parameters, such as pitch, jitter, shimmer, and hoarseness, were evaluated before and after surgery. The duration of the surgery was also compared.

**Results:**

The Mini-Window method significantly improved sound quality, resulting in lower voice shimmer and shorter surgery duration.

**Conclusion:**

While both methods are effective, the Mini-Window technique offers a more efficient surgery with reduced voice shimmer.

## Introduction

The glottis is the larynx part containing the vocal cords and the glottic opening (1). Anatomically, the glottis is a section of the larynx that extends from where the true and false vocal cords meet at the top of the laryngeal ventricle to a line one centimeter below the lower edge of the vocal cords. (2). 

The vocal cords play a key role in sound production, and their paralysis, unilaterally or bilaterally, can disrupt all or part of the laryngeal functions (3). A functional larynx is crucial for proper sound production and airway protection. (4). 

The recurrent laryngeal nerve damage causes hemi-larynx with decreased vocal cord tension, reduced mobility, and volume. It often leads to glottic insufficiency and dysphonia with a tendency to aspiration (5). 

Glottis insufficiency is the inability or failure of the structure of the larynx and vocal cords to produce sound (6). This condition can present with various clinical symptoms, such as blurred speech, fatigue when speaking, dysphagia and difficulty swallowing, decreased tone, and hoarseness (7). Glottic insufficiency occurs when the vocal folds fail to close completely during phonation, causing excessive air leakage through the glottis and increasing the risk of aspiration (8). Glottic insufficiency can result from various causes, the most common being vocal fold paralysis (complete immobility) or vocal fold paresis (weakness or partial immobility). Vocal fold immobility is considered a physical sign rather than a diagnosis. (9). 

Other causes of glottic insufficiency include vocal fold nodules or tumors, central or peripheral neurological injuries, sulcus vocalis, presbylaryngis, infections, intubation-related trauma, arytenoid injury, scarring or deformation of the vocal fold, among others. These causes may be congenital, idiopathic, or from iatrogenic factors, such as glottic tumor removal. (8).

Achieving better glottic closure during phonation is the main goal in managing glottic insufficiency. The glottic insufficiency could be treated with several surgical techniques. Arytenoid adduction, Medialization thyroplasty, reinnervation procedures, and vocal fold injection are popular among these techniques. Satisfactory voice results have been observed in all these methods for managing glottic insufficiency. However, the choice of surgical method must depend on the cause of glottic insufficiency, the etiologic factors of the patient, and some other situational factors (10).

One of the invasive treatments for repairing the atrophy of vocal cords or its paralysis due to various causes is the medialization laryngoplasty technique (11). 

Medialization laryngoplasty aims to restore vocal cord function and improve voice quality. In addition to the classical approach, a newer technique, the Mini-Window method, has been proposed, which involves a smaller incision.

This study aimed to evaluate and compare the effectiveness and surgical duration of medialization laryngoplasty with Gore-tex in two medialization laryngoplasty techniques, classic and the Mini window‌ method, in the periphery of false vocal cords.

## Materials and Methods

### Study design and patient selection:

This case series study was conducted from 2019 to 2021 at Rasool Akram Hospital, affiliated with the Iran University of Medical Sciences, Tehran, Iran, and included 30 patients with unilateral vocal cord paralysis. The study was approved by the Scientific and Ethics Committee of the Tehran University of Medical Sciences (IR.IUMS.FMD.REC.1399.602).

After obtaining written informed consent from participants, demographic information, including age, sex, occupation, and location, was collected using a structured checklist. All patients underwent comprehensive physiological, neurological, and psychological examinations.

The inclusion criteria for this study were patients diagnosed with unilateral vocal cord paralysis due to various causes, such as surgery, metastasis, or intubation, who showed no abnormalities in physiological, neurological, or psychological assessments. 

Exclusion criteria included patients with severe systemic diseases, individuals under 18 years of age, those with peripheral neuromuscular disorders, and patients with a history of neck surgeries that might affect laryngeal nerve function.

Participants were divided equally into two groups, with 15 patients in each group undergoing classic or mini-window techniques.

### Diagnostic Tools:

Laryngoscopy and stroboscopy were used to visually assess vocal fold movement, while acoustic analysis was performed to measure voice quality parameters. These evaluations were conducted one month before and one and six months after surgery.

### Surgical Techniques:

**Classic Medialization Laryngoplasty:**

 After making a 5 cm incision on the skin covering the thyroid ala, the strap muscles were turned to the sides, and the flap perichondrial was pulled upwards. Lower thyrotomy was performed 5 mm above the lower bridge of the thyroid cartilage. A 5 mm coarse diamond burr created the 10×6 mm2 surgical window. The internal perichondrium was then identified and pulled up, and then an incision was made along its lower and posterior margins. Using a laryngoscope, Gore-Tex was carefully placed on the gluteal surface to elevate the medial and posterior surface of the patient’s glottis. The perichondrium was then returned to its original location and put on the site of the thyrotomy.

**Mini-Window Method:**

After intravenous sedation and anesthesia injection in the form of subcutaneous in 4 quadrant laminas on the side of the incision surgery, a 3 cm long incision was made on the platysma and SCM muscles. Then, the thyroid notch and thyroid borders were exposed. After cutting the sternocleidomastoid muscle, it was removed from the lamina by retaining or removing the internal perichondrium and then opened at the perimeter of the 2×2 mm window of the vocal cords (preferably by preserving the internal perichondrium) without damaging the mucosa. Gore-Tex was placed on the outer surface of the inner perichondrium. After Gore-tex placement, sound overcorrection was performed, and after complete homeostasis, the site of strap and platysma muscle surgery was sutured with 0-4 absorbable sutures. The incised skin was sutured with 0-3 sutures, and the patient was discharged after receiving oral antibiotics.

**Outcome Measures:**

The primary outcomes were improved sound quality, assessed via parameters like jitter, shimmer, hoarseness, and breathiness. The secondary outcome was the duration of the surgery.

### Statistical analysis:

SPSS software was used for analysis. The Kolmogorov-Smirnov test was used to test the normality, and the Durbin-Watson test checked the independent error assumption. Baseline voice parameters were compared between the two groups to ensure data matching.

## Results

The mean age of patients was 50.32 ± 13.99 years in the classic medialization laryngoplasty group and 49.30 ± 12.62 years in the Mini-Window group. Baseline voice parameters (pitch, jitter, shimmer, hoarseness) were similar between the two groups, confirming matching between the groups before surgery. The results showed that surgery with the classical medialization laryngoplasty significantly improved patients’ sound factors. The difference between all audio factors before and after surgery was significantly reduced (P <0.05) (Table 1). 

**Table 1:** Results of sound analysis in the classical Medialization laryngoplasty surgical group

**Specifications of vocal cords**	**Pre-OP**	**Post-OP**	**P value**
Mean Pitch	133.02±33.5	125.06±31.02	0.003
Maximum Pitch	156.8±32.2	150.1±32.0	0.002
Voice Breaks	3	0	0.001
Voice Jitteriness	3.15±1.43	2.05±1.59	0.001
Voice Shimmer	1.10±0.675	0.902±0.704	0.04
Hoarseness	2.6±0.2	1.0±0.6	0.001
Breathy voice	2.9±0.5	1.7±0.8	0.001
SNR	17.50±3.38	13.02±4.10	0.002

Based on the statistical analysis results, the group that underwent reconstructive surgery by the Mini-Window method also showed similar results to classical surgery (P <0.05) (Table 2). 

**Table 2:** Results of sound analysis in the surgical group by Mini-Window method

**Specifications of vocal cords**	**Pre-OP**	**Post-OP**	**P value**
Mean Pitch	135.03±30.5	124.06±31.02	0.823
Maximum Pitch	155.3±30.1	149.1±32.0	0.902
Voice Breaks	6	0	0.506
Voice Jitteriness	3.14±1.20	2.05±1.43	0.080
Voice Shimmer	1.15±.616	1.02±0.704	0.120
Hoarseness	2.7±0.3	1.0±0.01	0.001
Breathy voice	2.8±0.9	1.4±0.8	0.052
SNR	16.92±3.53	12.05±4.10	0.053

Overall, 80% of patients experienced a significant enhancement in voice quality following surgery. Comparison of sound analysis results after surgery with the two classic Medialization laryngoplasty and Mini-Window methods showed that the difference between them was statistically significant only regarding voice shimmer (P =0.04). This factor decreased more in the Mini Window method than in the classical method, and no statistically significant difference was observed between other factors (Table 3).

**Table 3:** Comparison of sound analysis results after surgery in classical and Mini-Window methods

**Specifications of vocal cords**	**Post-OP Classic**	**Post-OP Mini-window**	**P value**
Mean Pitch	125.06±31.02	124.06±31.02	0.836
Maximum Pitch	150.1±32.0	149.1±32.0	0.602
Voice Breaks	0	0	1
Voice Jitteriness	2.05±1.59	2.05±1.43	0.733
Voice Shimmer	0.902±0.704	1.02±0.704	0.040
Hoarseness	1.0±0.6	1.0±0.01	0.630
Breathy voice	1.7±0.8	1.4±0.8	0.532
SNR	13.02±4.10	12.05±4.10	0.520

Statistical analysis of patients’ data obtained from completing the VHI questionnaire showed no significant difference in general, functional, physical, and emotional indices between the surgical groups by classical and mini-Window methods (Table 4).

**Table 4:** Results of the VHI (Voice Handicap Index) questionnaire

**P value**	**Mann-WhitneyU**	**Median (IQR)**		
		Mini window	Medialization laryngoplasty	Participating group
0.143	4963.50	25.00(15.00-40.00)	26.00 (14.50–40.00)	Total
0.236	4857.50	7.00(4.00-9.00)	8.00 (4.75–14.00)	Functional
0.152	4320.62	7.00(4.75.00-8.00)	8.00 (5.00–13.25)	Physical
0.142	4125.35	7.00(3.00-9.00)	8.00 (5.00–13.00)	Emotional

The average duration of surgery in the Mini-Window technique was significantly less than the classical method of Medialization laryngoplasty (P =0.0403) (Table 5).

**Table 5:** Duration of the study using the two methods of classical and Mini-Window

**P value**	**Time**	**Surgical procedure**
0.0403	6.5 minutes	Mini window
	10 minutes	Classic Medialization laryngoplasty

## Discussion

This study demonstrated that classical medialization laryngoplasty and the Mini-Window method effectively improved sound quality in patients with unilateral vocal cord paralysis. The Mini-Window technique had the additional benefit of reducing surgery time and shimmer. In medialization laryngoplasty, a lateral thyroid cartilage window is made at the vocal fold level, and an implant is placed in the paraglottic space to move the vocal fold tissue toward the glottic midline to improve vocal quality and efficiency (12). 

This method has become popular because it corrects the most common cause of vocal cord failure, paralysis. In a study by Tsai et al. (13), 35 patients with glottis insufficiency who underwent medialization laryngoplasty were studied. Before and after the study, all patients were evaluated for sound quality. Surgery results did not show a significant difference between men and women among the different groups (13). Over the years, various materials have been used for injection in the medialization laryngoplasty technique (14). One uses Gore-Tex tape implants, which have a flexible and soft structure, are easy to place and adjust during surgery, do not cause much inflammatory reaction, and can easily be removed (15). Gore-Tex is a Teflon product placed in the paraglottic space (16,17). 

Surgical procedures have been developed to restore the desired glottal position for phonetics. This study showed that the classic surgical techniques of Medialization laryngoplasty and Mini-Window improved the sound quality in patients, and they only differed in voice shimmer. These findings align with previous studies, such as those by Tsai et al. (13) and Wolter et al. (18), although the latter was conducted in pediatric populations. Wolter et al. examined the technique of medialization laryngoplasty surgery in children with unilateral motor dysfunction of the vocal cords in 38 patients who underwent surgery (18). Their studies showed that 78% of patients had better sound quality and 81% better swallowing quality than in the past (18). As in our study, patients’ voice quality and factors such as Voice Jitteriness and Shimmer improved significantly compared to the past.

## Conclusion

Both the classical and Mini-Window laryngoplasty techniques effectively improve voice quality in patients with unilateral vocal cord paralysis. The Mini-Window method offers the additional advantage of shorter surgery time and reduced voice shimmer. Future studies should investigate long-term outcomes and explore the potential benefits of other materials for medialization. While the Mini-Window method offers a more efficient procedure, it should be noted that individual patient factors (e.g., anatomical differences) may influence the choice of technique.
